# Necrosis by sodium overload-associated genes TRPM4 and SLC9A1: biological roles and clinical implications in breast cancer progression

**DOI:** 10.3389/fimmu.2025.1623511

**Published:** 2025-10-29

**Authors:** Yingze Zhu, Yaxin Guo, Yanlin Su, Zhuoqi Zhang, Yige Lu, Xianghan Zhang, Hui Pang

**Affiliations:** ^1^ The Fourth Department of Medical Oncology, Harbin Medical University Cancer Hospital, Harbin, Heilongjiang, China; ^2^ Capital Medical University, Beijing, China

**Keywords:** sodium-induced cell death, breast cancer, biomarkers, ion channel dysregulation, immunohistochemistry

## Abstract

**Background:**

Breast cancer persists as a principal contributor to global cancer mortality, driven by heterogeneous molecular pathways. Necrosis by sodium overload, a recently characterized form of regulated cell death, remains underexplored in oncogenesis. This study investigates the pathobiological significance and therapeutic potential of NECSO-related genes in breast cancer, elucidating their mechanistic roles in tumor progression.

**Methods:**

Multi-omics analyses were performed using transcriptomic data from TCGA and GEO cohorts (*n* = 1, 217), we systematically evaluated seven NECSO-related genes. Advanced bioinformatics pipelines included differential expression analysis, immune subtype profiling, functional state correlation, protein interaction mapping, and survival analytics. Experimental validation involved immunohistochemical evaluation of clinical samples.

**Results:**

Through multi-omics analysis of GEO and TCGA cohorts, we identified two sodium homeostasis-related genes, TRPM4 and SLC9A1, as consistently upregulated oncogenes in breast cancer, with significant diagnostic and prognostic relevance. Functional *in vitro* assays demonstrated that knockdown of either gene not only suppressed proliferation, colony formation, migration, and induced apoptosis in breast cancer cells, but also led to reduced expression of the sodium-calcium exchanger NCX1.

**Conclusion:**

TRPM4 and SLC9A1 is a novel prognostic biomarker and potential therapeutic target in breast cancer. Dysregulated sodium homeostasis mediated by NECSO-related genes represents a targetable vulnerability in precision oncology.

## 1 Introduction

Breast cancer (BC) remains the most prevalent malignancy among women worldwide, accounting for the highest incidence and mortality rates in numerous regions (1). Despite advancements in early screening, a subset of patients presents with aggressive or metastatic disease at diagnosis (2, 3), underscoring the urgent need for improved therapeutic strategies. Current treatments, including chemotherapy, endocrine therapy, and HER2-targeted regimens, have enhanced survival outcomes, yet drug resistance and recurrence persist as major challenges (4). Consequently, identifying novel biomarkers for early diagnosis and prognosis is critical. Recently, emerging research has highlighted sodium dysregulation as a driver of a unique cell death pathway—NECSO—characterized by sodium ion overload disrupting mitochondrial energetics and membrane integrity (5, 6).

Elevated intracellular sodium levels are observed in BC tissues compared to normal breast epithelium (7), and sodium channel inhibitors have demonstrated antitumor effects in preclinical models. NECSO is a form of regulated cell death triggered by sustained intracellular sodium ([Na^+^]_j_) overload, leading to fatal disruption of cellular ion homeostasis, membrane potential, and osmotic balance (8). Unlike mere osmotic lysis, NECSO involves specific ion channels and transporters and is characterized by a sustained rise in [Na^+^]_j_ that precedes cell death and can be pharmacologically inhibited. It is distinct from apoptosis, as it occurs independently of caspase-3/7 activation and lacks classic apoptotic morphology such as membrane blebbing and apoptotic body formation. Furthermore, NECSO differs fundamentally from ferroptosis (which is iron-dependent) and pyroptosis (mediated by gasdermin), as it is primarily driven by sodium-specific ionotoxicity.

Intriguingly, sodium homeostasis-related genes, including TRPM4 and SLC9A1, are aberrantly expressed in BC and correlate with metastasis and poor survival (9, 10). Although sodium-based compounds show therapeutic potential (11), the prognostic and diagnostic roles of NECSO-related genes in BC remain underexplored, with most evidence derived from computational studies. This study identified TRPM4 and SLC9A1 as NECSO-related differentially expressed genes through integrated analysis of GEO datasets. Multi-omics validation was performed using TCGA, METABRIC, and CPTAC databases, followed by immunohistochemical assays to evaluate protein expression and clinical relevance in BC cohorts. Our findings reveal the diagnostic and prognostic utility of TRPM4 and SLC9A1 in BC, offering insights into sodium dysregulation as a therapeutic target.

## 2 Materials and methods

### 2.1 Sample information

Emerging evidence implicates sodium dysregulation as a pivotal mechanism in sodium overload-induced necrosis, a distinct cell death modality characterized by cytotoxic Na^+^ accumulation and mitochondrial membrane collapse. Guided by prior mechanistic studies delineating ion transport pathophysiology, we prioritized seven evolutionarily conserved sodium regulators—NC1, CLT, DHPs, SLC12A2 (NKCC1), SLC8A1 (NCX1), TRPM4, and SLC9A1 (NHE1)—based on their established roles in maintaining transmembrane sodium gradients. To investigate their dysregulation patterns in breast carcinogenesis, we leveraged two orthogonal transcriptomic repositories: The GSE42568 dataset from GEO provided microarray-based expression profiles, while the TCGA-BRCA cohort (*n* = 1, 226; Illumina HiSeq RNA-seq) furnished paired raw count data and clinicopathological annotations (AJCC Stage I-IV). This dual-cohort approach enabled cross-platform validation of sodium homeostasis-associated transcriptional alterations, with TCGA data subjected to TPM normalization and combat batch correction to minimize technical variability.

### 2.2 Differential gene expression analysis

Bioinformatic analyses were conducted in R (v4.2.1) using rigorously validated computational pipelines. Raw microarray data from the GSE42568 dataset were retrieved via the GEOquery package (v2.68.0) and subjected to probe-level quality control: Multi-mapped probes were systematically filtered, retaining only the highest intensity probe per gene using the collapseRows algorithm (WGCNA v1.72). Variance-stabilizing transformation (VST) implemented in DESeq2 (v1.38.0) normalized count distributions prior to differential expression analysis, with statistical significance determined by Wald test (|log_2_FC| >1, *p* < 0.05). Parallel processing of TCGA-BRCA RNA-seq data (STAR-aligned, TPM-normalized) incorporated generalized linear models through the stats package (v4.2.1) with empirical Bayes variance moderation. Dimensionality reduction and visualization workflows generated comparative boxplots, principal component analysis (PCA) biplots, and volcano plots using ggplot2 (v3.4.0) with ggrepel (v0.9.3) for label optimization.

### 2.3 Receiver operator characteristic curve and survival analysis

Diagnostic performance evaluation incorporated receiver operating characteristic (ROC) curve analysis implemented in the pROC package (v1.18.0). For TRPM4 and SLC9A1, time-dependent AUC values were calculated to assess discriminative capacity between malignant and non-malignant specimens, with statistical significance determined via DeLong’s test for paired ROC curve comparisons. Survival analytics employed Cox proportional hazards regression models (survival v3.3.1) to evaluate sodium dysregulation-associated mortality risk, incorporating molecular subtype stratification and age-adjusted multivariate analyses. Kaplan-Meier curves with log-rank *P*-values were generated using survminer, featuring time-to-event endpoints censored. Hazard ratios and 95% confidence intervals were computed through restricted mean survival time comparisons between high/low expression cohorts defined by X-tile optimized cutpoints.

### 2.4 Baseline data table

TRPM4 and SLC9A1 expression cohorts were stratified to determine optimal survival-discriminatory thresholds. Baseline clinicopathological characteristics between high/low expression groups were compared using multivariate logistic regression models, incorporating Fisher’s exact tests for categorical variables and Welch’s *T*-tests for continuous parameters (age, tumor size). Missing clinical data were imputed via chained equations with 10 iterations, while inverse probability weighting adjusted for potential selection bias. All statistical tests were two-sided with Bonferroni correction applied to address multiplicity.

### 2.5 Functional enrichment analysis and gene set enrichment analysis

Selected patients were divided into two groups based on TRPM4 and SLC9A1 expression, and target genes were extracted by utilizing package “DESeq2” difference analysis with |log_2_(FC)|> 0.5 and adj*P* value < 0.05 as screening criteria. (GO) including biological process (BP), cellular component (CC), molecular function (MF) and (KEGG) pathway enrichment analyses were performed using package “clusterProfiler [4.4.4]” (21), *P* < 0.05 was considered statistically significant. Gene Set Enrichment Analysis (GSEA) was conducted using the “clusterProfiler” package, and the top 4 enriched terms were displayed in a mountain plot. Te GSEA results were visualized using the “ggplot2” package.

### 2.6 Construction of mRNA−miRNA regulatory network

We utilized the miRNet database (https://www.mirnet.ca/) to predict the association between diferentially expressed mRNAs and miRNAs. Subsequently, an mRNA-miRNA regulatory network was constructed based on the obtained results.

### 2.7 Protein–protein interaction network analyses of TRPM4 and SLC9A1

Potential protein interactions with TRPM4 and SLC9A1 were collected and integrated through the STRING database (https://string-db.org/) and extracted the relevant genes from these interactions to conduct PPI network analysis.

### 2.8 Clinical data collection

Paraffin-embedded breast cancer tissues and paired adjacent normal tissues were collected from 90 patients diagnosed with breast cancer via histopathology at Harbin Medical University Cancer Hospital between January 2010 and December 2024. Inclusion criteria: (1) Histopathologically confirmed invasive breast carcinoma; (2) No prior neoadjuvant therapy, chemotherapy, or radiotherapy before surgery; (3) Age range 18–70 years; (4) At least one measurable lesion confirmed by imaging (mammography, ultrasound, or MRI); (5) Normal hematological, hepatic, renal, and cardiac function parameters. For deceased patients, the cause of death was directly attributed to breast cancer progression. Exclusion criteria: (1) Age <18 or >70 years; (2) Severe comorbidities; (3) History of other malignancies; (4) Preoperative anticancer treatments (targeted therapy, immunotherapy); (5) Incomplete clinical follow-up data. Written informed consent was obtained from all participants, and the study protocol was approved by the Ethics Committee of Harbin Medical University Cancer Hospital.

### 2.9 Immunohistochemical testing

Te specimens were fxed in formalin, dehydrated, embedded, and sectioned into 4-μm continuous slices. Immunohistochemical analysis was performed using the MaxVision method. The TRPM4 and SLC9A1 antibody was purchased from ProteinTech; Te universal immunohistochemistry MaxVision kit and DAB staining solution were purchased from Proteintech. Te working concentration of the TRPM4 antibody was 1:100, and the working concentration of the SLC9A1 antibody was 1:200. Te specifc staining steps were strictly followed according to the instructions provided with the kit.

### 2.10 Western blotting

Protein content was analyzed with a Bicinchoninic Acid Protein Assay Kit (Thermo Fisher Scientific, Waltham, USA). The protein samples were separated with sodium dodecyl sulfate-polyacrylamide gel electrophoresis, and the protein bands were transferred to PVDF membranes (03010040001, Roche). Membranes were probed overnight at 4°C using primary antibodies against the relevant proteins. After primary antibody removal via washing, secondary antibodies were introduced for a 1-hour incubation period at room temperature. Protein band visualization was achieved through chemiluminescent detection with the Abbkine superkine™ ECL kit (China), imaged on the Tanon system (China).

### 2.11 Statistical analysis

Statistical analysis was performed using one-way ANOVA to compare the means across different groups. All statistical tests were performed with GraphPad Prism software (version 9; GraphPad Software, La Jolla, CA, USA). Data are presented as means ± standard deviations (SDs), as indicated in the figure legends. A *P*-value of less than 0.05 was considered statistically significant.

## 3 Results

### 3.1 Differential expression genes of NECSO related genes in normal tissues and breast cancer tissues based on GEO database analysis

**Figure 1:**
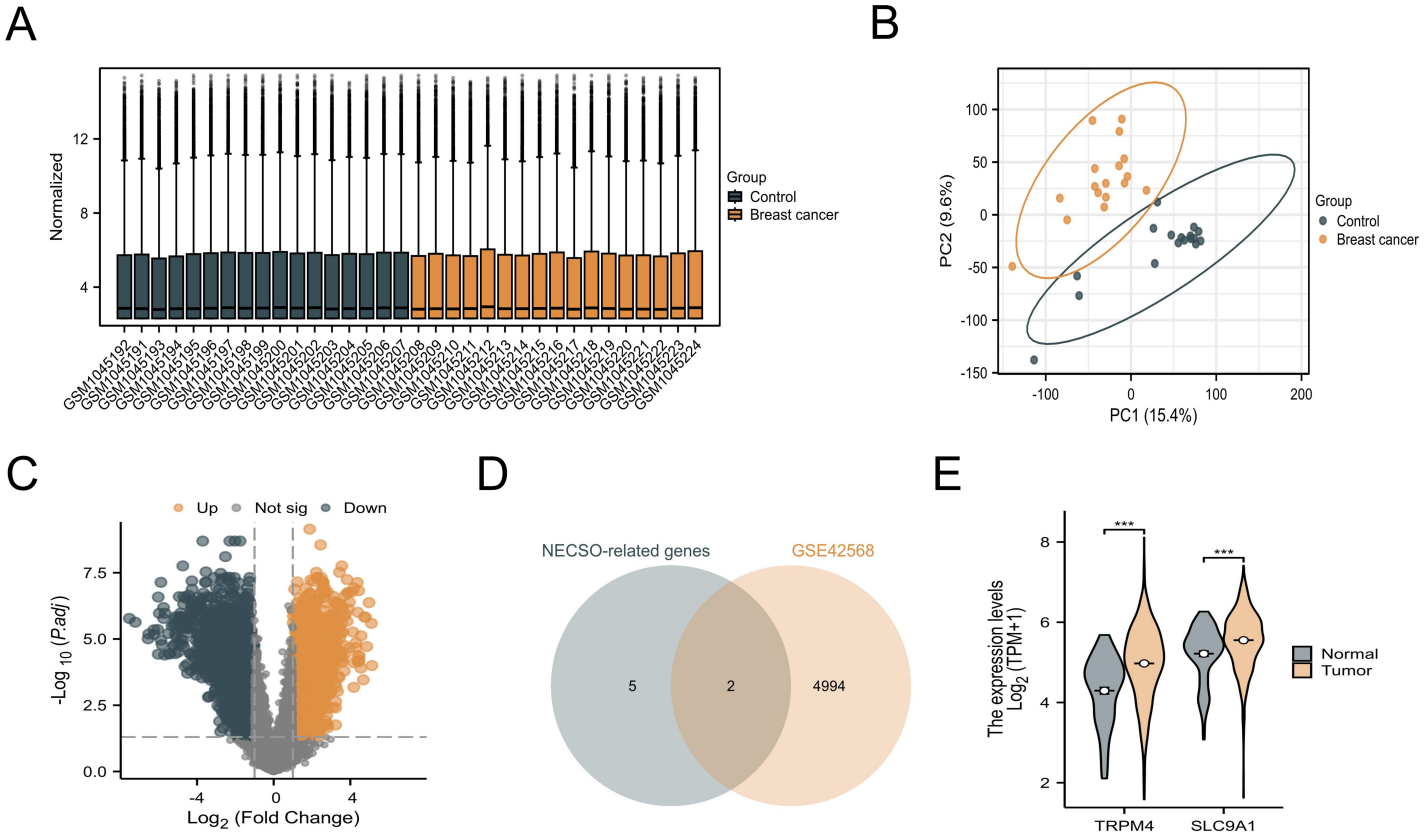
Identification and validation of NECSO-associated genes. **(A)** VST-normalized expression heatmap of the GSE42568 dataset. **(B)** Principal component analysis plot demonstrating transcriptional divergence in GSE42568. **(C)** Volcano plot of differentially expressed genes in GSE42568. **(D)** Comparative expression analysis of TRPM4 and SLC9A1 between normal mammary tissues and breast carcinoma specimens from GSE42568 ***P<0.001.

Our investigation of NECSO-associated genes in breast carcinogenesis commenced with systematic analysis of the GSE42568 dataset from GEO. Raw microarray data underwent normalization using variance-stabilizing transformation (VST) via DESeq2 (v1.38.0) to mitigate technical variability (**Figure 1A**). Principal component analysis (PCA) revealed distinct clustering patterns between tumor and normal cohorts (**Figure 1B**), confirming substantial transcriptomic divergence. Differential expression analysis identified 4, 996 genes meeting stringent thresholds (|log_2_FC| > 0.5, *p* < 0.05) through DESeq2’s Wald test, visualized as a volcano plot (**Figure 1C**). From seven candidate NECSO regulators previously implicated in sodium homeostasis (NC1, CLT, DHPs, SLC12A2, SLC8A1, TRPM4, SLC9A1), intersection analysis identified two NECSO-associated DEGs - TRPM4 and SLC9A1 - showing consistent tumor-specific upregulation (**Figure 1D**). Cross-validation using TCGA-BRCA RNA-seq data (*n* = 1, 098 tumors vs. 113 normals) confirmed significant overexpression of both genes in malignant tissues (**Figure 1E**).

### 3.2 miRNA target prediction and diagnostic validation

**Figure 2:**
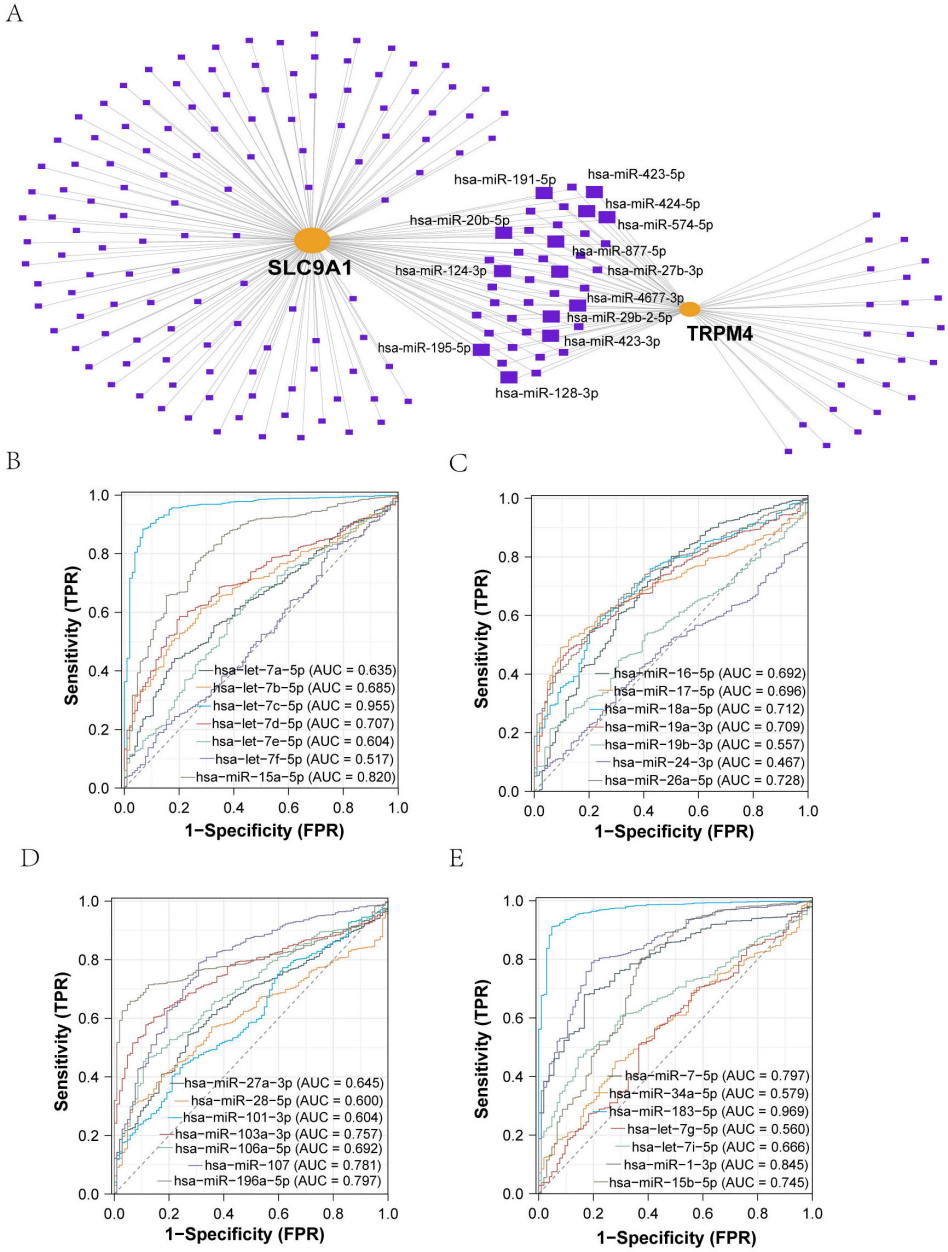
Identification and Diagnostic Evaluation of miRNAs Targeting TRPM4 and SLC9A1. **(A)** Prediction of common miRNAs associated with sodium death-related genes and their diagnostic value: TRPM4 and SLC9A1 interacting miRNAs were predicted via an mRNA-miRNA regulatory network. **(B-E)** ROC analysis of miRNAs identified through PubMed search, all showing certain diagnostic value.

We employed the miRNet database (v2.0) to predict miRNAs regulating TRPM4 and SLC9A1, identifying 28 shared candidates (**Figure 2A**), including hsa-miR-106a-5p, hsa-miR-18a-5p, hsa-miR-101-3p, hsa-miR-16-5p, hsa-miR-27a-3p, hsa-miR-24-3p, hsa-miR-26a-5p, hsa-miR-17-5p, hsa-miR-19a-3p, hsa-let-7e-5p, hsa-miR-19b-3p, hsa-let-7d-5p, hsa-miR-28-5p, hsa-let-7c-5p, hsa-miR-15b-5p, hsa-let-7i-5p, hsa-let-7b-5p, hsa-let-7g-5p, hsa-miR-34a-5p, hsa-miR-1-3p, hsa-miR-7-5p, hsa-miR-196a-5p, hsa-miR-107, hsa-let-7a-5p, hsa-miR-183-5p, and hsa-miR-15a-5p. Predictions integrated TargetScan (v7.2) and miRDB (v6.0) algorithms with a cumulative interaction weight >0.95. Diagnostic evaluation of these miRNAs in the TCGA-BRCA cohort (*n* = 1, 098) revealed hsa-let-7c-5p and hsa-miR-183-5p as top discriminators between malignant and normal tissues (**Figures 2B-E**). Literature validation through systematic PubMed mining confirmed experimental evidence for all 28 miRNAs in BC pathogenesis, with hsa-miR-34a-5p, hsa-let-7c-5p, and hsa-miR-183-5p showing direct tumor-suppressive roles.

### 3.3 Analysis of TRPM4 and SLC9A1 in breast cancer based on TCGA

**Figure 3:**
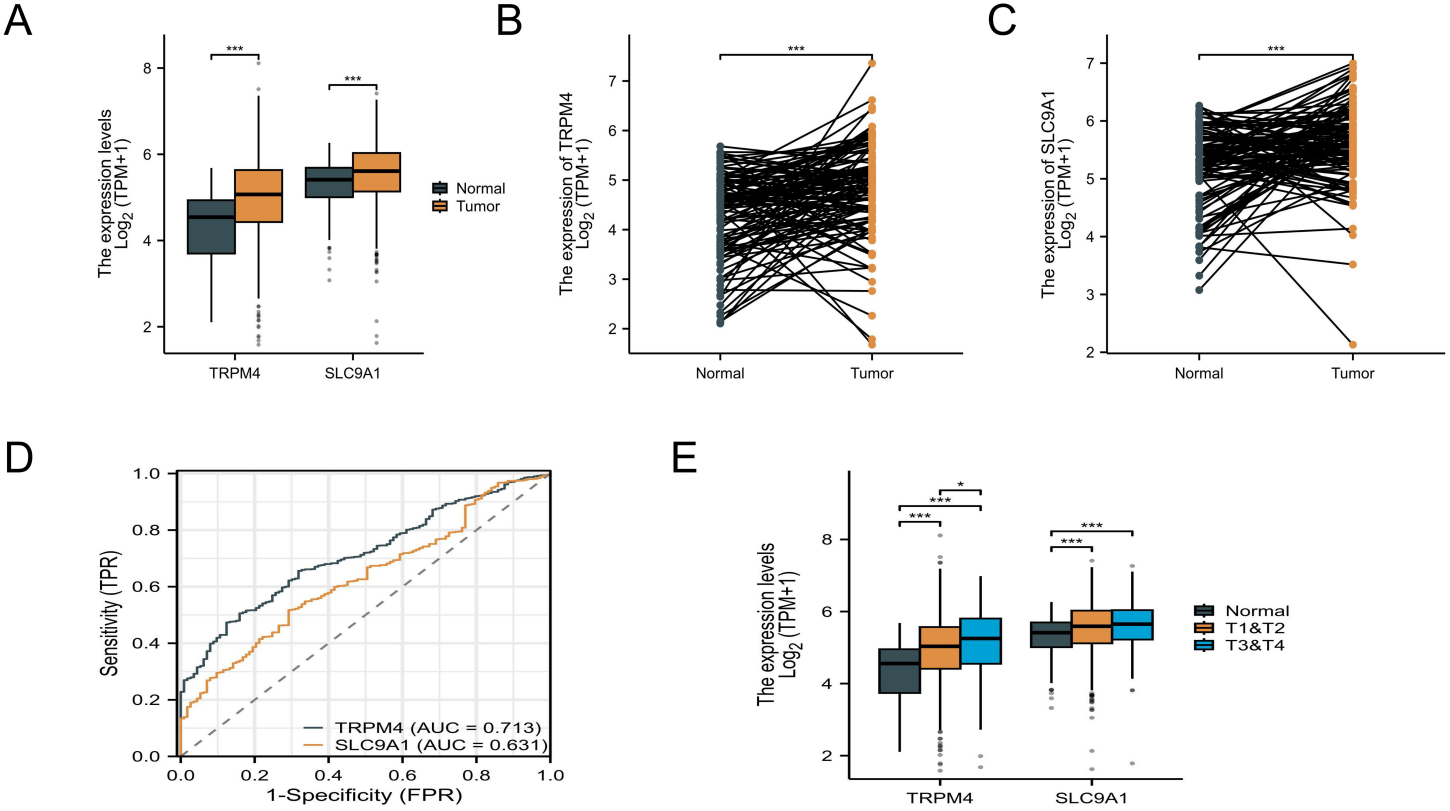
Pan-cancer and BC expression analysis. **(A)** Unpaired sample analysis showing upregulated expression of TRPM4 and SLC9A1 in BC compared to normal tissues. **(B, C)** Paired sample analysis demonstrating upregulated TRPM4 and SLC9A1 expression in BC versus adjacent normal tissues. **(D)** ROC curve analysis indicating high diagnostic value for TRPM4 and low diagnostic value for SLC9A1 in BC. **(E)** Expression levels of TRPM4 and SLC9A1 across different tumor stages *P<0.05 ***P<0.001.

By integrating TCGA and GTEx data, TRPM4 was found to exhibit the highest expression level in PRAD, while SLC9A1 showed the highest expression in ESCA (**Supplementary Figures 1A, B**). Compared to normal tissues, TRPM4 and SLC9A1 expression levels were significantly upregulated in BRCA samples (*P* < 0.05), with statistical significance, as shown in **Figure 3A**. In paired samples, both TRPM4 and SLC9A1 were upregulated in BRCA patients (**Figures 3B, C**). An ROC curve constructed using the BRCA dataset from the TCGA database (**Figure 3D**) demonstrated high diagnostic value for TRPM4. Additionally, TRPM4 and SLC9A1 were associated with tumor staging: their levels were upregulated in T3-T4 stage BRCA patients compared to T1-T2 stage patients (**Figure 3E**). The expression of TRPM4 in BRCA tissues was significantly associated with overall survival (OS, *P* = 0.01) and disease-specific survival (DSS, *P* = 0.008), but showed no significant correlation with progression-free interval (PFI, *P* = 0.052). In contrast, the protein expression levels of SLC9A1 and TRPM4 demonstrated no significant prognostic relevance (**Supplementary Figures 2A-F**). COX proportional hazards regression identified TRPM4, as an independent prognostic biomarker in breast cancer. In univariate analysis, high TRPM4 expression was significantly associated with reduced mortality risk (*HR* = 0.691; 95% *CI*: 0.498-0.958; *P* = 0.027), whereas SLC9A1 expression showed no association with prognosis (*HR* = 1.097; 95% *CI*: 0.797-1.509; *P* = 0.571). After adjusting for clinical covariates, TRPM4 retained its independent protective effect (*HR* = 0.691; 95% *CI*: 0.498-0.958; *P* = 0.027), confirming its robust prognostic value. Notably, SLC9A1 failed to demonstrate significance in multivariate analysis, suggesting its expression is confounded by other clinical factors. These findings highlight TRPM4 as a promising biomarker for risk stratification in breast cancer, while SLC9A1 warrants further investigation to elucidate its biological role (**Supplementary Figure 2G**).

### 3.4 Genetic alterations of TRPM4 and SLC9A1

**Figure 4:**
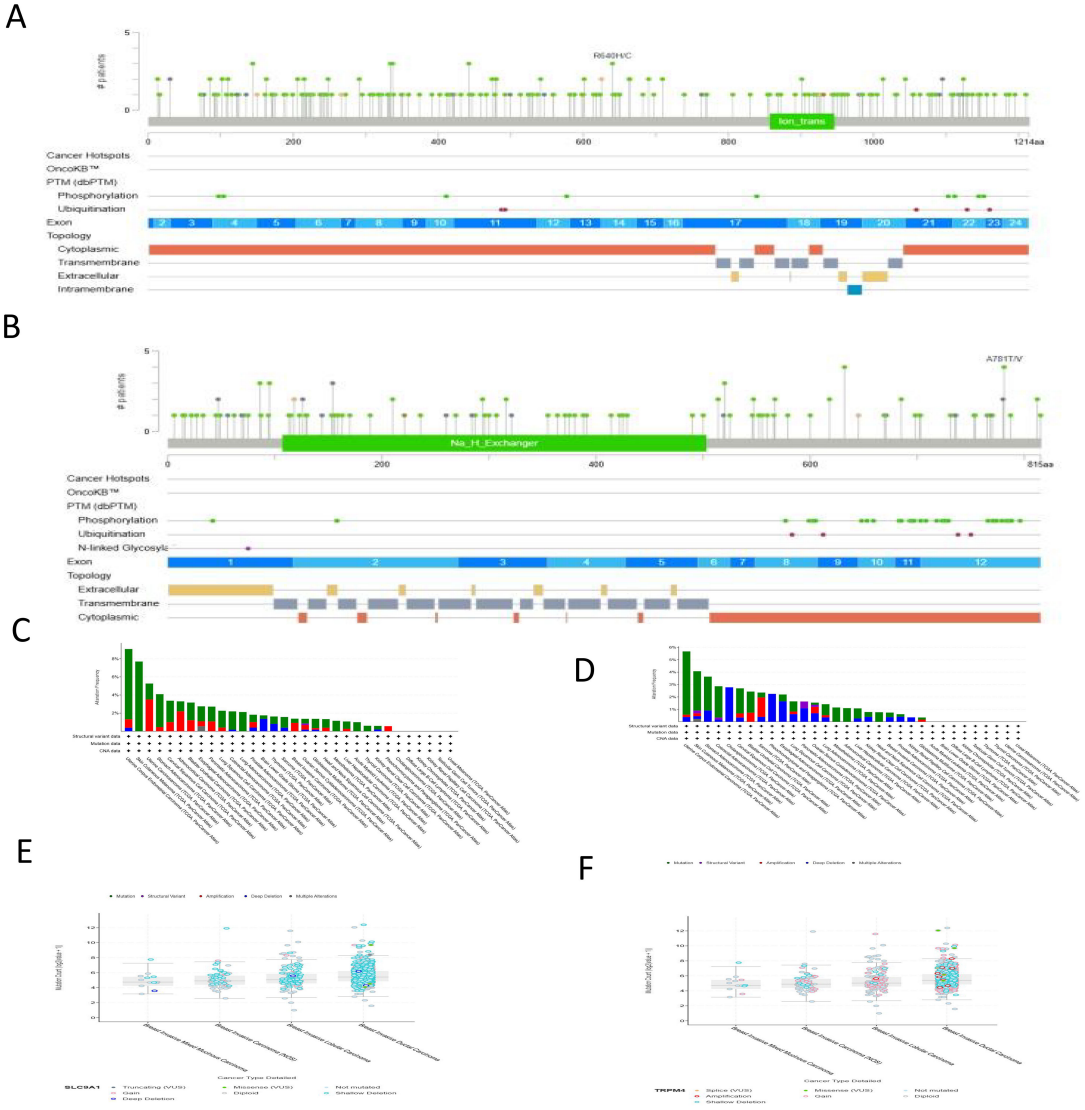
Mutation Analysis of TRPM4 and SLC9A1. (**A, B**) Mutation distribution maps of TRPM4 and SLC9A1 across their protein structural domains. **(C)** TRPM4 mutation landscape based on the TCGA pan-cancer atlas. **(D)** SLC9A1 mutation landscape based on the TCGA pan-cancer atlas. **(E)** Higher mutation counts of SLC9A1 in BIDC compared to BIMM. **(F)** Higher mutation counts of TRPM4 in BIDC compared to BIMM.

**Figure 5:**
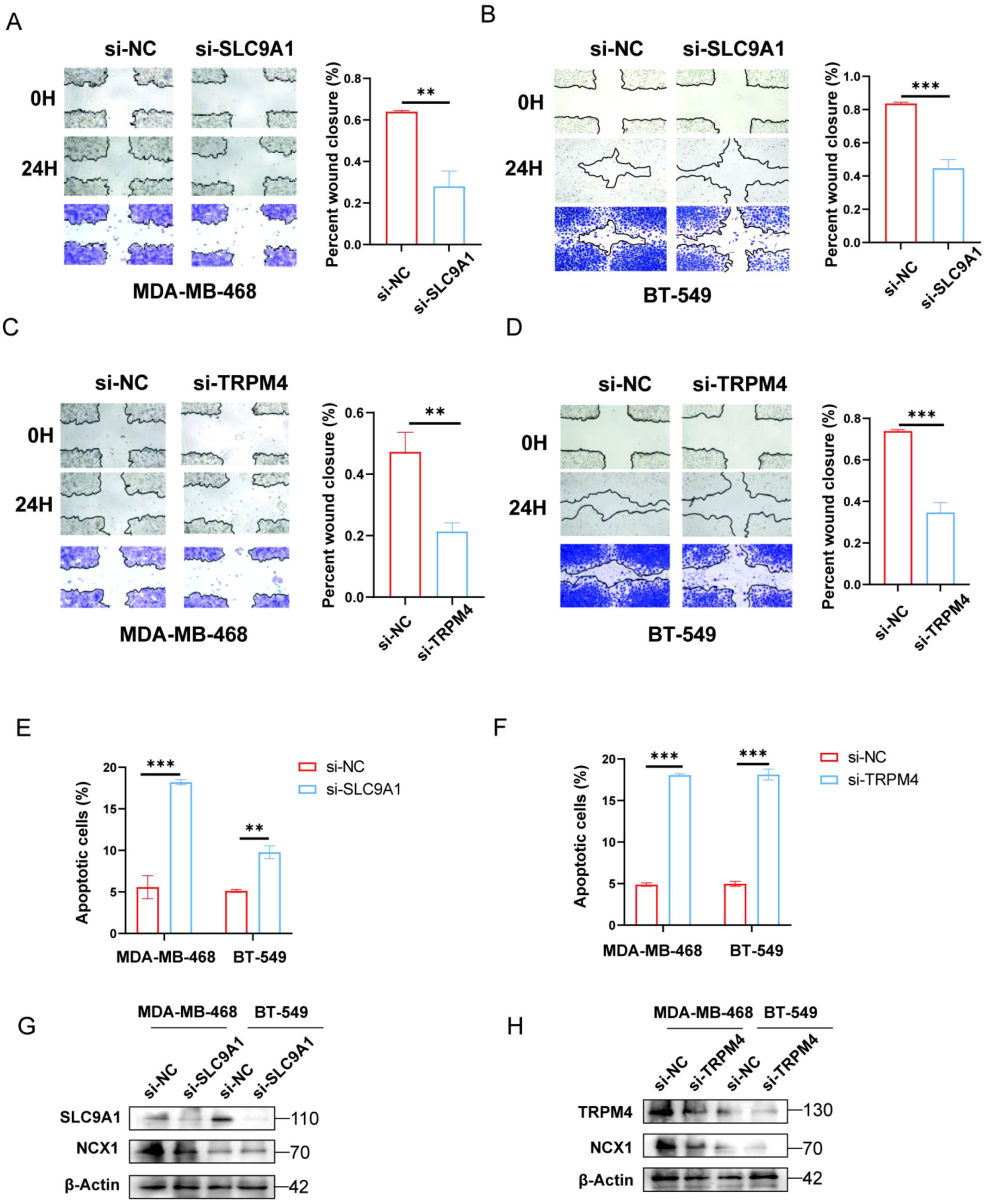
Effects of TRPM4 and SLC9A1 knockdown on migration, apoptosis, and NCX1 expression in cancer cells. **(A-D)** Transwell assay was performed to assess the migration of MDA-MB-468 and BT-549 cells transfected with si-NC, si-TRPM4, and si-SLC9A1. **(E, F)** Flow cytometry was used to detect apoptosis in MDA-MB-468 and BT-549 cells after knockdown of TRPM4 and SLC9A1. **(G, H)** Western blot analysis was conducted to examine the effect of TRPM4 and SLC9A1 knockdown on the protein expression level of NCX1 **P<0.01 ***P<0.001.

Analysis of genetic alterations in TRPM4 and SLC9A1 across cancers was performed using the cBioPortal platform, encompassing 32 studies and 10, 967 samples (**Figures 4A, B**). TRPM4 harbored 12 mutation sites, including 49 missense mutations, 8 in-frame mutations, with R80Q being the most frequent mutation type. SLC9A1 exhibited 9 mutation sites, predominantly missense mutations, along with truncations, splice variants, structural variants (SVs)/fusions, and A781TV as the most common mutation type. Mutations in TRPM4 and SLC9A1 were observed in 32 high-grade breast cancer tissues (**Figures 4C, D**). TRPM4 mutations may impair calcium ion permeability, leading to intracellular sodium/calcium imbalance and promoting tumor migration. Missense mutations in SLC9A1 could disrupt sodium-hydrogen exchange function, exacerbating tumor microenvironment acidification and enhancing chemotherapy resistance. Although somatic mutations in TRPM4 and SLC9A1 are relatively rare events in breast cancer overall (**Figures 4E, F**), the specific mutations that do occur may offer valuable functional insights. For instance, TRPM4 mutations could potentially impair its calcium ion permeability, which might contribute to intracellular sodium/calcium imbalance—a phenomenon supported by our functional data in knockdown models (**Figures 5G, H**). Similarly, missense mutations in SLC9A1 could disrupt sodium-hydrogen exchange, a mechanism that might exacerbate microenvironment acidification. Interestingly, we observed that these mutations were even less frequent in invasive mixed mucinous carcinoma compared to invasive ductal carcinoma, suggesting a distinct genetic landscape. However, given their low prevalence, their contribution to the overall breast cancer population is likely limited. Therefore, the primary clinical relevance of TRPM4 and SLC9A1 in breast cancer is more robustly underscored by their frequent mRNA/protein overexpression and strong association with poor prognosis, as demonstrated throughout our study, rather than by their mutational status.

### 3.5 PPI network and enrichment analysis

**Figure 6:**
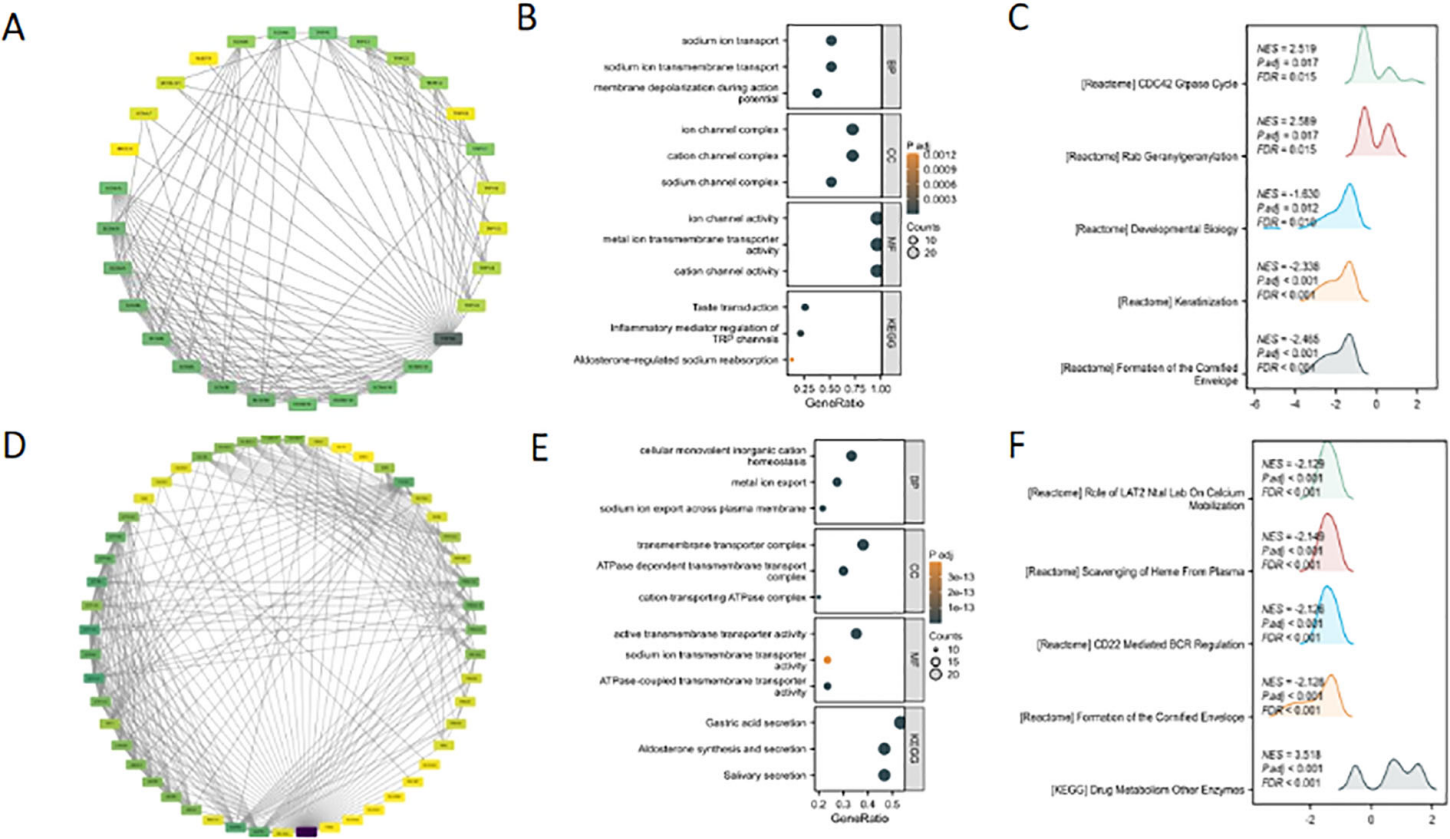
Bioinformatic analysis of TRPM4 and SLC9A1: PPI networks and functional enrichment. **(A, D)** PPI network of TRPM4 and SLC9A1. **(B, E)** GO/KEGG pathway enrichment analysis of TRPM4 and SLC9A1 interacting proteins. **(C, F)** Top 5 enriched pathways from GSEA functional enrichment analysis of TRPM4 and SLC9A1.

The protein-protein interaction (PPI) networks of TRPM4 and SLC9A1 were constructed using a high-confidence threshold (score ≥ 0.7) with the top 30 interacting proteins (**Figures 6A, D**). GO/KEGG enrichment analysis of their interacting proteins revealed that TRPM4 was significantly associated with sodium ion transport, sodium ion transmembrane transport-mediated membrane depolarization, ion channel complexes (including cation/sodium channel complexes), and metal ion transmembrane transporter activity (**Figures 6B, E**), while its KEGG pathway enrichment primarily involved taste transduction. GSEA further demonstrated that TRPM4 was enriched in pathways such as the CDC42 GTPase Cycle, Rab Geranylgeranylation, Developmental Biology, and Keratinization (**Figures 6C, F**). In contrast, SLC9A1’s GSEA results highlighted pathways including the Role of LAT2/NTAL in Calcium Mobilization, Scavenging of Heme from Plasma, CD22-Mediated BCR Regulation, Formation of the Cornified Envelope, and Drug Metabolism by Other Enzymes.

### 3.6 Functional states of TRPM4 and SLC9A1 in breast cancer via the CancerSEA database

**Figure 7:**
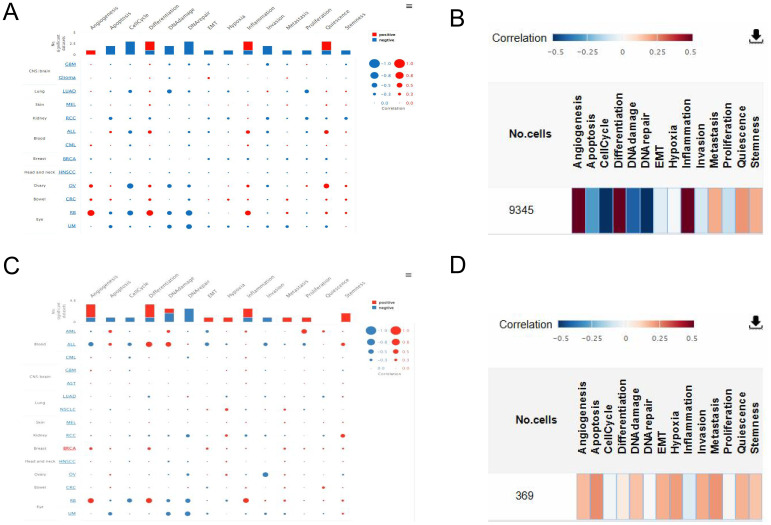
Correlation of TRPM4 and SLC9A1 with Tumor Functional States. **(A)** Interactive bubble plot showing correlations between TRPM4 and functional states across 16 cancer types. **(B)** Interactive bubble plot showing correlations between SLC9A1 and functional states across 16 cancer types. **(C)** Correlation of TRPM4 with functional states in BC. **(D)** Correlation of SLC9A1 with functional states in BC.

The functional states of TRPM4 and SLC9A1 in breast cancer were investigated using the CancerSEA database (**Figures 7A, B**). TRPM4 exhibited strong positive correlations with angiogenesis (**Figure 7C**), while SLC9A1 showed positive correlations with DNA repair, DNA damage, and cell cycle regulation (**Figure 7D**).

### 3.7 Expression levels of TRPM4 and SLC9A1

**Figure 8:**
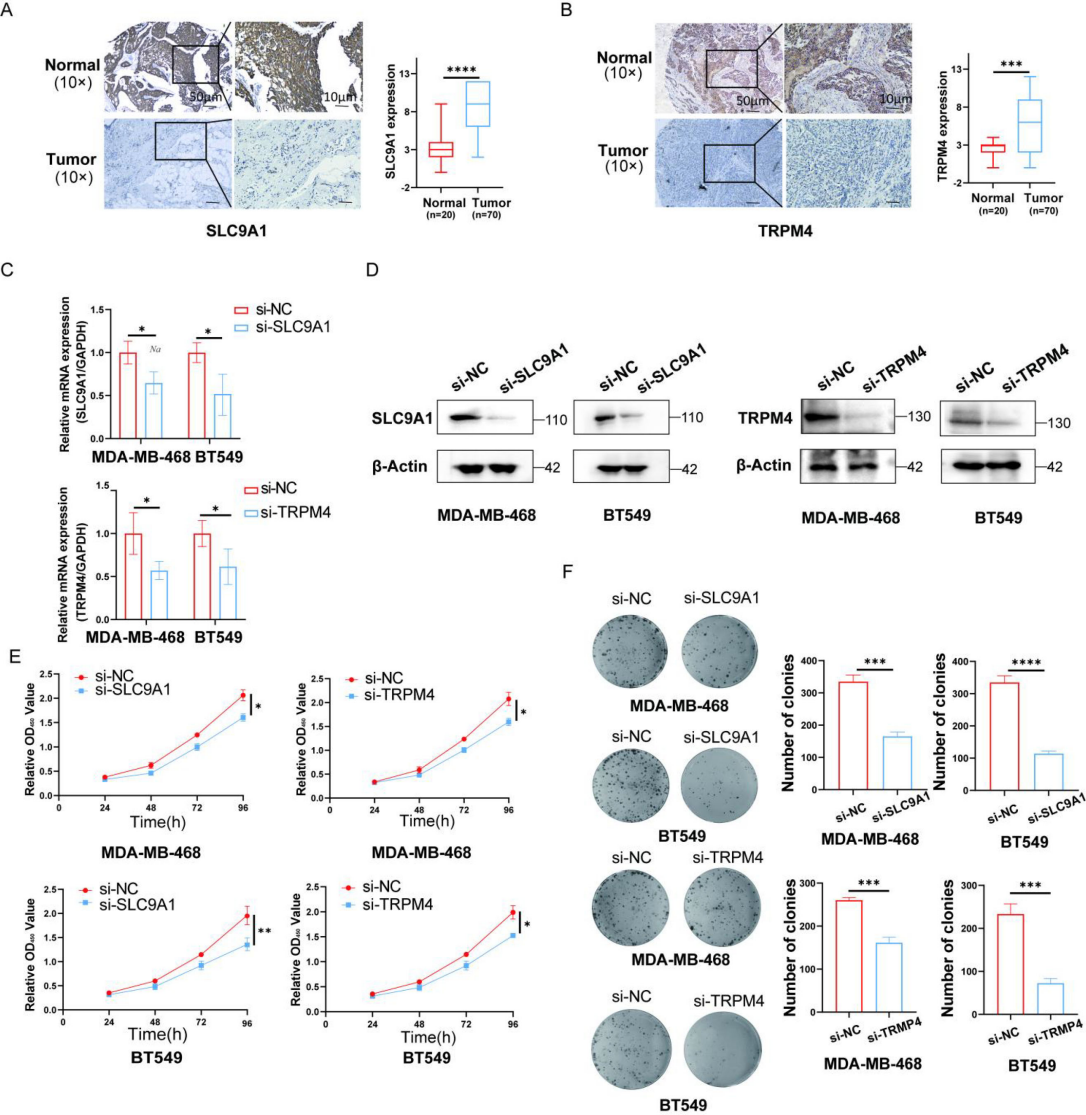
Knockdown of TRPM4 and SLC9A1 Suppresses Proliferation and Clonogenicity in Breast Cancer Cells. **(A, B)** Immunohistochemical analysis was performed to evaluate the expression of TRPM4 and SLC9A1 in tumor tissues (Scale bars, 50 μm). The knockdown efficiency of TRPM4 and SLC9A1 was confirmed by RT-qPCR. Western blot was used to assess the protein expression levels of TRPM4 and SLC9A1 in cells following knockdown. Cell viability after TRPM4 and SLC9A1 knockdown was measured using the CCK-8 assay. Colony formation assay was conducted to evaluate the clonogenic ability of BT-549 and MDA-MB-468 cells after knockdown of TRPM4 and SLC9A1. *P < 0.05, **P < 0.01, ***P < 0.001, ****P < 0.0001.

We employed immunohistochemistry to analyze the expression of TRPM4 and SLC9A1 in tumor tissues. The results revealed that the expression levels of TRPM4 and SLC9A1 were significantly upregulated compared to those in the normal tissue group (**Figures 8A, B**). Subsequently, we performed RT-qPCR and Western blot assays to validate the knockdown efficiency of si-TRPM4 and si-SLC9A1 (**Figures 8C, D**). We transfected MDA-MB-468 and BT-549 cells with si-RNAs targeting TRPM4 and SLC9A1, and conducted CCK-8 and colony formation assays to evaluate the proliferative capacity mediated by these genes. The colony formation assay demonstrated that the clonogenic ability was significantly reduced in the TRPM4 and SLC9A1 knockdown groups compared to the negative control group (**Figures 8E, F**).

Similarly, the scratch wound healing assay demonstrated that compared to the NC group, the cell migration ability was significantly reduced in the TRPM4 and SLC9A1 knockdown groups (**Figures 5A-D**). Subsequently, the effect of TRPM4 and SLC9A1 knockdown on cell apoptosis was detected by flow cytometry. It was found that the apoptosis rate was significantly higher in cells after knockdown of these two genes compared to the control group (**Figures 5E, F**). To further verify the correlation between these two genes and sodium channels, we examined the expression of NCX1, a core regulator of intracellular sodium and calcium ions. Western blot analysis showed that genetic knockdown of either TRPM4 or SLC9A1 significantly reduced the protein expression level of NCX1 (**Figures 5G, H**).

These results indicate that knockdown of TRPM4 and SLC9A1 significantly inhibits the proliferation and migration abilities of breast cancer cells, thereby suppressing tumor growth. Mechanistically, TRPM4 and SLC9A1 cooperatively regulate proliferative signaling through ion homeostasis and modulate metastatic capacity by affecting the epithelial-mesenchymal transition pathway, positioning them as novel therapeutic targets. This dual functionality, spanning tumorigenesis, progression, and microenvironmental adaptation, provides a pioneering framework for developing precision therapies against breast cancer, particularly for treatment-resistant subtypes.

## 4 Discussion

This study elucidates the pathogenic roles of TRPM4 and SLC9A1 in breast cancer progression. Both genes are overexpressed in BC and are associated with sodium overload-induced mitochondrial dysfunction, aligning with prior reports of dysregulated sodium homeostasis in cancer. TRPM4, a sodium-permeable channel, likely drives metastasis through calcium signaling dysregulation, while SLC9A1, a sodium-hydrogen exchanger, may acidify the tumor microenvironment to promote therapy resistance. The prognostic significance of SLC9A1 underscores its therapeutic potential, particularly in advanced BC. The miRNA regulatory network reveals post-transcriptional control of TRPM4/SLC9A1, with hsa-let-7c-5p and hsa-miR-183-5p emerging as diagnostic biomarkers. These findings corroborate the established tumor-suppressive role of let-7 family miRNAs in BC (12–16).

Despite the compelling evidence we have provided, several limitations of this study should be acknowledged. First, the sample size of our cohort (n=90), although prospectively stratified to ensure balanced representation across all major molecular subtypes, remains relatively modest. While this balanced design empowered robust cross-subtype comparisons, it may still limit the statistical power for more nuanced multivariate analyses or for investigating rare clinical subgroups within each subtype. Consequently, the generalizability of our findings warrants further validation in larger, independent cohorts that reflect the natural prevalence of breast cancer subtypes. Future multi-center studies with larger sample sizes are essential to confirm our results and to establish more definitive cutoff values for clinical application. Beyond the oncogenic functions of SLC9A1 and TRPM4 themselves, a key question remains regarding their upstream regulation. Our bioinformatic analysis suggests that their expression may be under the control of a network of tumor-suppressive miRNAs (hsa-let-7c-5p and hsa-miR-183-5p). Although the experimental validation of these specific interactions falls beyond the scope of the current study, which prioritized the functional characterization of the core sodium homeostasis axis, exploring this epigenetic layer of regulation represents a compelling future direction. Confirming these predictions will be essential to paint a complete picture of the regulatory hierarchy governing sodium-induced cell death in cancer.

First, the retrospective design of the analyses inherently restricts causal inference, as confounding variables may influence the observed associations between sodium dyshomeostasis-related genes and clinical outcomes. Second, while bioinformatics approaches provided robust correlative insights, the lack of functional validation in experimental models limits mechanistic clarity regarding how TRPM4 and SLC9A1 directly regulate sodium ion fluxes or interact with downstream oncogenic pathways. Third, the reliance on bulk transcriptomic data from public databases may mask tumor heterogeneity, including spatial variations in sodium homeostasis within the tumor microenvironment or subclonal genetic alterations affecting sodium channel activity. Fourth, the clinical cohorts analyzed lack detailed longitudinal sodium-level measurements or pharmacological interventions targeting sodium transporters, precluding an assessment of dynamic sodium dysregulation during BC progression. Future studies should integrate multi-omics profiling with functional assays to delineate the precise roles of TRPM4/SLC9A1 in sodium-driven tumorigenesis and validate their therapeutic relevance in prospective cohorts (17–20). In conclusion, TRPM4 and SLC9A1 serve as critical regulators of sodium homeostasis in BC, exhibiting both diagnostic and prognostic relevance. SLC9A1 represents a promising biomarker for aggressive disease. Targeting sodium dysregulation pathways may offer novel therapeutic strategies to mitigate BC progression and chemoresistance (21–24).

## Data Availability

The original contributions presented in the study are included in the article/**Supplementary Material**. Further inquiries can be directed to the corresponding author.
