# Supplementary figures and images for: Necrosis by sodium overload-associated genes TRPM4 and SLC9A1: biological roles and clinical implications in breast cancer progression
Source: Front Immunol. 2025 Oct 29;16:1623511. doi: 10.3389/fimmu.2025.1623511 (PMC12605240; doi:10.3389/fimmu.2025.1623511)

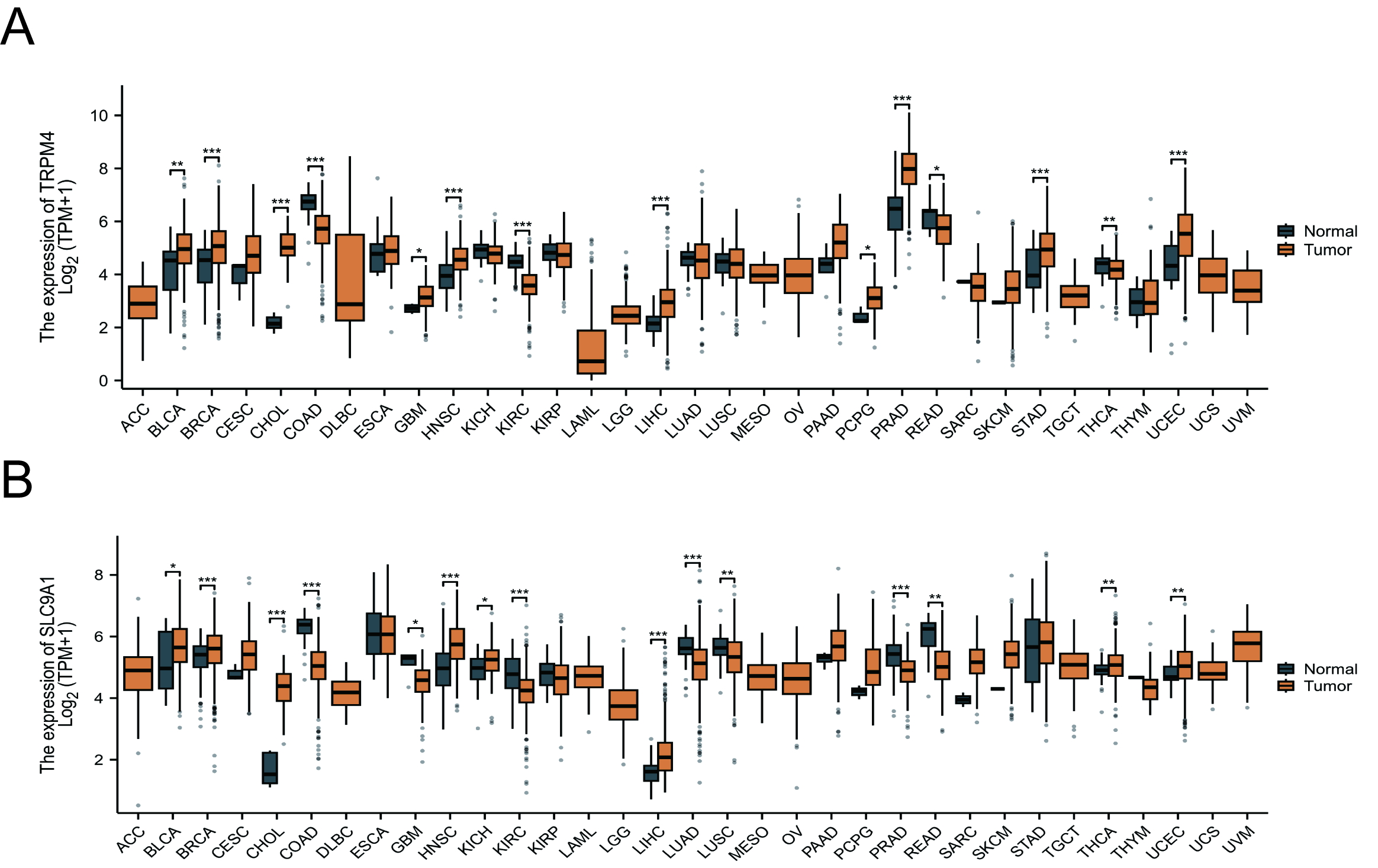

Supplement: Supplementary file 1 [file Image1.tif]

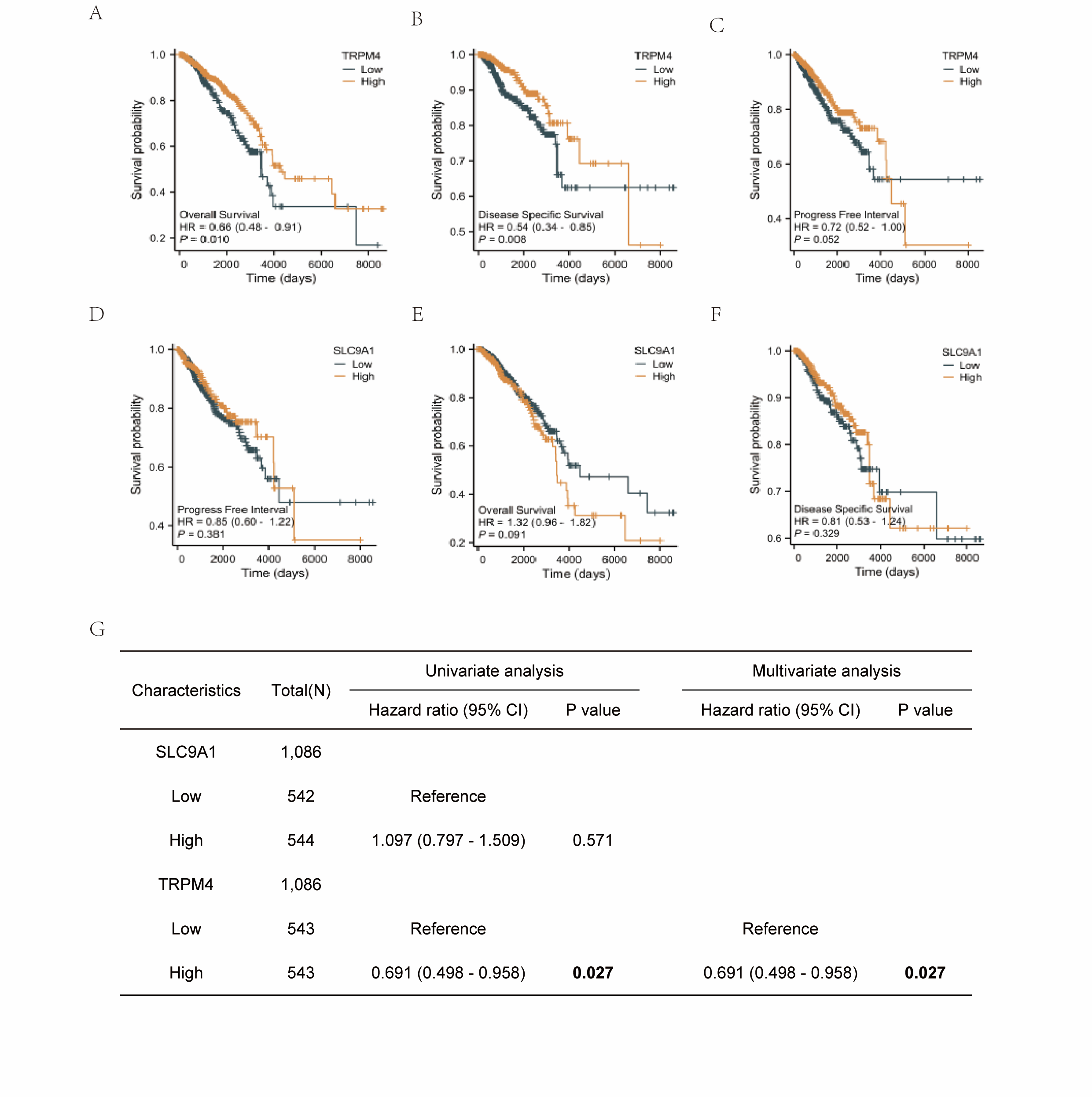

Supplement: Supplementary file 2 [file Image2.png]
